# Sedentary behavior and subclinical atherosclerosis in African Americans: cross-sectional analysis of the Jackson heart study

**DOI:** 10.1186/s12966-016-0349-y

**Published:** 2016-03-01

**Authors:** Keith M. Diaz, John N. Booth, Samantha R. Seals, Steven P. Hooker, Mario Sims, Patricia M. Dubbert, Paul Muntner, Daichi Shimbo

**Affiliations:** Department of Medicine, Center for Behavioral Cardiovascular Health, Columbia University Medical Center, 622 West 168th Street, PH9-319, New York, NY 10032 USA; Department of Epidemiology, School of Public Health, University of Alabama at Birmingham, Birmingham, AL USA; Center of Biostatistics and Bioinformatics, University of Mississippi Medical Center, Jackson, MS USA; School of Nutrition and Health Promotion, Arizona State University, Phoenix, AZ USA; Department of Medicine, University of Mississippi Medical Center, Jackson, MS USA; South Central Mental Illness, Research, and Clinical Center and Little Rock Geriatric Research, Education, and Clinical Center, Little Rock, AR USA

**Keywords:** Sedentary, Television viewing, Atherosclerosis, African Americans, Carotid intima–media thickness

## Abstract

**Background:**

Previous studies have reported conflicting results as to whether an association exists between sedentary time and cardiovascular disease (CVD) risk among African Americans. These studies, however, were limited by lack of consideration of sedentary behavior in leisure versus non-leisure settings. To elucidate this relation, we investigated the associations of television (TV) viewing time and occupational sitting with carotid intima-media thickness (CIMT), a subclinical atherosclerosis measure, in a community-based sample of African Americans.

**Methods:**

We studied 3410 participants from the Jackson Heart Study, a single-site, community-based study of African Americans residing in Jackson, MS. CIMT was assessed by ultrasonography and represented mean far-wall thickness across right and left sides of the common carotid artery. TV viewing time, a measure of leisure sedentary behavior, and occupational sitting, a measure of non-leisure sedentary behavior, were assessed by questionnaire.

**Results:**

In a multivariable regression model that included physical activity and CVD risk factors, longer TV viewing time (2–4 h/day and >4 h/day) was associated with greater CIMT (adjusted mean ± SE difference from referent [<2 h/day] of 0.009 ± 0.008 mm for 2–4 h/day, and 0.028 ± 0.009 mm for >4 h/day; P-trend =0.001). In contrast, more frequent occupational sitting (‘sometimes’ and ‘often/always’) was associated with lower CIMT (adjusted mean ± SE difference from referent [‘never/seldom’]:−0.021 ± 0.009 mm for ‘sometimes’, and−0.018 ± 0.008 mm for ‘often/always’; P-trend = 0.042).

**Conclusions:**

Longer TV viewing time was associated with greater CIMT, while occupational sitting was associated with lower CIMT. These findings suggest the role of sedentary behaviors in the pathogenesis of CVD among African Americans may vary by whether individuals engage in leisure versus non-leisure sedentary behaviors.

**Electronic supplementary material:**

The online version of this article (doi:10.1186/s12966-016-0349-y) contains supplementary material, which is available to authorized users.

## Background

African Americans have higher rates of cardiovascular disease (CVD), cancer, and a shorter life expectancy compared with whites [1–3]. Rates of obesity and diabetes are continuing to increase disproportionately among African Americans, raising concern racial disparities in health outcomes will continue to grow [1, 4, 5]. As a result, there is a need to identify modifiable risk factors in African Americans amenable to behavioral intervention.

Sedentary behaviors are independent, modifiable risk factors for CVD outcomes and mortality even among individuals who meet physical activity recommendations [6]. As such, sedentary behaviors are now thought to represent a unique aspect of an individual’s overall physical activity profile and is no longer considered simply to be the extreme low end of the physical activity continuum [7]. The association between sedentary behaviors and CVD risk among African Americans, however, is unclear. Findings from the Coronary Artery Risk Development in Young Adults (CARDIA) study, National Health and Nutrition Examination Survey (NHANES), and Youth Risk Behavioral Survey have shown no association between sedentary behavior and CVD risk in African Americans [8–10]. More recently, sedentary behavior was associated with all-cause mortality, but not CVD mortality, among African Americans in the Southern Community Cohort Study [11]. Thus, there are conflicting findings regarding whether an association exists between sedentary behavior and CVD risk among African Americans. However, these previous studies were limited by relatively small sample sizes of African American participants and/or lack of consideration of sedentary behavior in leisure versus non-leisure settings. Occupational sedentary time has been reported to have no association or a less adverse association with CVD risk than leisure-based sedentary behaviors including television (TV) viewing [12–15]. Thus, assessing overall sedentary behavior as a summary measure across leisure and non-leisure domains could obscure associations.

The purpose of this study was to examine the associations of TV viewing and occupational sitting with carotid artery intima-media thickness (CIMT), a measure of subclinical atherosclerosis, in a large community-based sample of African Americans enrolled in the Jackson Heart Study (JHS). We secondarily investigated the association of occupational standing time with CIMT.

## Methods

### Study population

The JHS is a large, single-site, population-based study of CVD among African Americans. Details of study design, recruitment, and data collection have been previously described [16–19]. Briefly, 5301 non-institutionalized African American adults aged ≥ 21 years were enrolled between 2000 and 2004 from the Atherosclerosis Risk in the Community (ARIC) site in Jackson, Mississippi (30 %) and a regionally representative sample of urban and rural residents from the Jackson, Mississippi metropolitan tri-county region (Hinds, Madison and Rankin counties) that were randomly contacted (17 %), volunteers (22 %), or secondary family members (31 %). Data for the current cross-sectional analysis were collected during the baseline examination period which included an in-home interview and a clinical examination after an overnight fast. Information on sociodemographic characteristics, health behaviors, and medical history were collected via questionnaire during the in-home interview administered by trained African American interviewers. The clinical examination included blood sampling, anthropometric measurement, carotid ultrasonography, and blood pressure measurement to evaluate CVD risk factors. A pill bottle review of medications taken in the 2 weeks prior to the clinical examination was also conducted during the clinical examination. The median number of days between the in-home visit and the clinical examination was 13 days (25th–75th percentile: 7–26 days).

### Assessment of sedentary behaviors, standing time, and physical activity

Sedentary behaviors, standing time, and physical activity were assessed using the JHS Physical Activity Survey, a 30-item questionnaire administered during the in-home interview that assesses physical activity over the past 12 months. The JHS Physical Activity Survey was adapted from modifications to the Baecke physical activity survey and was designed to improve physical activity assessment in African Americans [20]. TV viewing was assessed using the single-item question: ‘During the past year, how often did you watch television?’ with response options of ‘Less than 1 h a week’, ‘At least 1 h a week, but less than 7 h a week’, ‘At least 1 h a day but less than 2 h a day’, ‘At least 2 h a day but less than 4 h a day’, and ‘four or more hours a day’. Occupational sitting was assessed using the single-item question: ‘When you are at work how often do you sit?’ with response options of ‘Never’, ‘Seldom’, ‘Sometimes’, ‘Often’, and ‘Always’. Time spent standing was also assessed in the occupational domain using the single-item question: ‘When you are at work how often do you stand?’ with response options of ‘Never’, ‘Seldom’, ‘Sometimes’, ‘Often’, and ‘Always’. The assessment of TV viewing using a single, close-ended, quantitative response item has been validated against accelerometry (rho = 0.22) and has good reproducibility (*r* = 0.75–0.78) [21, 22]. The assessment of occupational sitting using a single, close-ended response item has been validated against accelerometry supplemented with an activity log (rho = 0.63) and has moderate-to-good reproducibility (intraclass correlation coefficient = 0.74) [23].

Leisure-time moderate to vigorous physical activity (MVPA) was assessed by a series of questions related to the yearly frequency and weekly duration of participation in up to three sports or exercise in the past year. Responses were converted to “minutes per week” for each sport or exercise reported and incorporated the number of months a participant partook in the sport or exercise (minutes/week = [hours/week*60]*[months in past year/12 months]) as previously described [24]. Each sport/exercise was assigned a metabolic equivalent of task (MET) value using the Compendium of Physical Activities [25]. Levels of leisure-time MVPA were estimated by summing the minutes per week of participation in sports or exercise with a MET value ≥3.5. Minutes per week of leisure-time MVPA was heavily right skewed (46.9 % of participants reported 0 min of leisure-time MVPA). Accordingly, MVPA was expressed as a categorical variable to account for its non-normal distribution. Consistent with the American Heart Association’s “Life’s Simple seven” metric, participants were stratified as having poor (0 min of MVPA/week), moderate (>0 and <150 min of MVPA/week and >0 and <75 min of vigorous physical activity/week), or ideal (≥150 min of MVPA/week or ≥75 min of vigorous physical activity/week) levels of leisure-time MVPA [26]. Summary measures derived from the sport and exercise-related items of the JHS physical activity survey have been validated against MVPA objectively-measured by accelerometry (rho = 0.24) and have high reproducibility (intraclass correlation coefficient = 0.99) [27].

### Carotid ultrasonography

An electrocardiography-gated, B-mode, and spectral steered Doppler with an integrated recorder ultrasound machine was used to obtain the carotid artery images in a 7.5 MHz linear-array transducer [16]. Images were obtained bilaterally at the far and near walls on three segments of the carotid artery: (1) the common carotid artery (CCA), (2) bifurcation of the carotid artery, and (3) internal carotid artery. All segments were imaged from the optimal angle (the angle of interrogation that most clearly shows the separation of the internal and external carotid arteries and the tip of the flow divider). The observed values were obtained for each segment, side, and wall. For each segment, sequences of 150 consecutive frames over approximately five cardiac cycles were digitized. The widest diameter frame during systole was then selected for measurement based on visualization of arterial interfaces. Maximum likelihood estimates were calculated by adjusting for missing data in the collecting, processing, and reading of carotid images. CCA intima media thickness (CIMT) represented a maximum likelihood estimate of the average values across the right and left CCA far wall.

### Covariates

Sociodemographic characteristics (age, sex, education, income, employment status), selected CVD risk factors (body mass index [BMI], diabetes, hypertension, cholesterol [total, LDL, and HDL]), lifestyle behaviors (cigarette smoking, alcohol consumption, energy intake, physical activity), estimated glomerular filtration rate (eGFR), self-reported history of myocardial infarction, and statin use were included as covariates. A detailed summary of methodology for these variables are reported in Additional file 1.

### Statistical analyses

The current analysis was restricted to 3419 participants with complete data for TV viewing, occupational sitting/standing, and CIMT. Participants who either simultaneously reported sitting and standing at work as ‘never’ or reported both sitting and standing at work as ‘always’ were excluded (*n* = 9), leaving a final sample size of 3410. Characteristics of participants included and excluded from analyses are presented in Additional file 1: Table S1.

For analyses of TV viewing, participants were grouped as follows: <2, 2–4, and >4 h/day. Linear regression models were used to evaluate the association of TV viewing time with CIMT. Unadjusted regression models were first assessed modeling TV viewing (2–4 h/day and >4 h/day) as indicator variables with TV viewing time <2 h/day serving as the referent. Subsequent models included adjustment for age and sex (model 1), further adjustment for education less than high school, annual family income < $50,000, fulltime employment, heavy alcohol consumption, current smoking, energy intake, history of myocardial infarction, and statin use (model 2), additional adjustment for diabetes, hypertension, BMI, total cholesterol, HDL cholesterol, LDL cholesterol, and eGFR <60 ml/min/1.73 m^2^ (model 3), and finally adjustment for MVPA category (model 4) and occupational sitting (model 5). Pairwise comparisons and linear trends across TV viewing categories were evaluated. The above analyses were then repeated in a fully adjusted model testing interactions for age (<60 and ≥60 years), sex (male and female), and MVPA category (poor, moderate, or ideal) by including an interaction term in the regression model. To account for missing data among covariates, all multivariate models included only cases with complete covariate data in the most adjusted model (e.g., model 5).

All of the above analyses were repeated to examine the association of occupational sitting and, separately, occupational standing with CIMT (with adjustment for TV viewing in the most adjusted model [model 5]). For both occupational sitting and standing, participants were grouped as sitting/standing ‘never or seldom’, ‘sometimes’, and ‘often or always’, with ‘never or seldom’ serving as the referent. Data analyses were conducted using SPSS version 22 (SPSS Inc, Chicago, IL).

### Ethics, consent and permissions

The JHS adhered to the guidelines set forth by the Declaration of Helsinki and was approved by institutional review boards of participating institutions. All participants provided informed consent.

## Results

### Participant characteristics

Among the 3410 participants available for analysis, 1111 (32.6 %), 1247 (36.6 %), and 1052 (30.9 %) participants watched TV <2 h/day, 2–4 h/day, and >4 h/day, respectively. Participants who reported longer daily TV viewing were, on average, older, more likely to have an annual family income < $50,000 and less likely to complete high school and be full-time employed (Table 1).

**Table 1:** Characteristics of JHS participants (*n* = 3410) by category of television viewing

	Television viewing			
Variable	<2 h/day	2–4 h/day	>4 h/day	P-Trend
	*(n = 1111)*	*(n = 1247)*	*(n = 1052)*	
Age (years)	52.8 ± 12.7	53.2 ± 12.4	54.7 ± 13.1	<0.001
Male sex (%)	36.5	37.4	37.8	0.534
Education < HS (%)	15.4	16.5	20.3	0.003
Income < $50,000 (%)	68.0	67.0	72.6	0.023
Fulltime Employment (%)	56.8	57.6	50.9	0.006
Heavy alcohol drinking (%)^a^	4.0	4.1	3.2	0.330
Current Smoking (%)	13.6	11.4	13.8	0.945
Energy Intake (kcal/d)	2235.5 ± 1360.6	2228.1 ± 1229.8	2209.5 ± 1227.9	0.890
History of MI (%)	4.9	4.6	4.9	0.934
Statin Use (%)	10.5	10.6	12.1	0.245
Diabetes (%)^b^	18.3	19.2	21.4	0.069
Hypertension (%)^c^	55.4	60.8	59.3	0.058
BMI (kg/m^2^)	31.5 ± 7.0	32.1 ± 7.4	31.8 ± 7.6	0.133
Obese (%)	51.6	56.5	52.6	0.579
Total Cholesterol (mg/dL)	197.6 ± 39.5	199.0 ± 41.5	201.0 ± 40.3	0.178
HDL Cholesterol (mg/dL)	51.9 ± 14.6	51.4 ± 14.4	51.5 ± 14.5	0.638
LDL Cholesterol (mg/dL)	125.9 ± 37.4	126.3 ± 36.9	129.0 ± 36.6	0.139
eGFR <60 ml/min/1.73 m^2^ (%)	5.6	5.0	6.2	0.559
Leisure-time MVPA (min/week)	72.6 ± 117.0	70.4 ± 112.6	67.2 ± 111.3	0.547
Level of MVPA (%)^d^				0.265
Poor	45.9	45.8	49.2	
Intermediate	33.1	33.5	30.2	
Ideal	21.0	20.7	20.5	

Data presented as mean ± standard deviation or percentage

*HDL* high density lipoprotein, *HS* high school, *MI* myocardial infarction, *MVPA* moderate to vigorous physical activity

^a^Defined as >14 drinks/week for men; >7 drinks/week for women

^b^Defined as fasting glucose ≥126 mg/dL, HbA1c ≥6.5 %, or use of diabetic medication

^c^Defined as blood pressure ≥140/90 mmHg and/or use of antihypertensive medication

^d^Defined according to American Heart Association Life’s Simple 7 criteria for minutes/week of moderate or vigorous physical activity. Poor physical activity: 0 min/week of leisure-time moderate or vigorous physical activity. Intermediate physical activity: >0 and <150 min/week of leisure-time moderate physical activity; and >0 and <75 min/week of leisure-time vigorous physical activity. Ideal physical activity: ≥150 min/week of leisure-time moderate physical activity; or ≥75 min/week of leisure-time vigorous physical activity

For occupational sitting, 945 (27.7 %), 988 (29.0 %), and 1477 (43.3 %) participants reported sitting at work ‘never or seldom’, ‘sometimes’, or ‘often or always’, respectively. Participants sitting at work were, on average, younger, more likely to be full-time employed, and less likely to be using statins (Additional file 1: Table S2). Participant characteristics across occupational standing categories are presented in Additional file 1: Table S3.

### TV Viewing and CIMT

In unadjusted models, longer daily TV viewing was associated with greater CIMT (Table 2). This association remained statistically significant in all adjusted models (models 1–5, Table 2). The association between TV viewing and CIMT did not vary by age, sex, or MVPA category (all interaction *p*-values >0.05; data not shown).

**Table 2:** Differences in CIMT associated with television viewing categories

	Television viewing					
	<2 h/day	2–4 h/day	>4 h/day			
	*(n = 1417)*	*(n = 1699)*	*(n = 1885)*	P1	P2	P-Trend
CIMT (mm)	0.715 ± 0.187	0.726 ± 0.184	0.749 ± 0.195			
Unadjusted	1 (ref)	0.010 ± 0.007	0.027 ± 0.008	0.192	<0.001	<0.001
Model 1	1 (ref)	0.009 ± 0.008	0.028 ± 0.009	0.287	0.001	0.001
Model 2	1 (ref)	0.008 ± 0.008	0.028 ± 0.009	0.307	0.001	0.001
Model 3	1 (ref)	0.009 ± 0.008	0.028 ± 0.009	0.275	0.001	0.001
Model 4	1 (ref)	0.009 ± 0.008	0.028 ± 0.009	0.273	0.001	0.001
Model 5	1 (ref)	0.009 ± 0.008	0.028 ± 0.009	0.258	0.001	0.001

Data presented as mean ± standard deviation or unadjusted/adjusted mean difference compared to referent group (<2 h/day) ± standard error

P1 = 2–4 h/day vs. <2 h/day (ref); P2= >4 h/day vs. <2 h/day (ref)

*CIMT* carotid intima-media thickness

Model 1: Adjusted for age and sex

Model 2: Adjusted for covariates in model 1 plus education < high school, income < $50,000, fulltime employment, heavy alcohol drinking, current smoking, energy intake, history of myocardial infarction, and statin use

Model 3: Adjusted for covariates in model 2 plus diabetes, hypertension, BMI, total cholesterol, HDL cholesterol, LDL cholesterol, and estimated glomerular filtration rate <60 ml/min/1.73 m^2^

Model 4: Adjusted for covariates in model 3 plus levels of moderate to vigorous physical activity (poor, intermediate, or ideal)

Model 5: Adjusted for covariates in model 4 plus occupational sitting category (never/seldom, sometimes, or often/always)

### Occupational sitting and CIMT

In unadjusted models, more frequent occupational sitting was associated with lower CIMT (Table 3, Upper Panel). This association remained statistically significant in all adjusted models (models 1–5, Table 3). The association between occupational sitting and CIMT did not vary by age, sex, or MVPA category (all interaction *p*-values >0.05; data not shown).

**Table 3:** Differences in CIMT associated with occupational sitting (upper panel) and occupational standing (lower panel) categories

	Occupational sitting					
	Never or seldom	Sometimes	Often or always			
	*(n = 945)*	*(n = 988)*	*(n = 1477)*	P1	P2	P-Trend
CIMT (mm)	0.728 ± 0.187	0.706 ± 0.174	0.711 ± 0.183			
Unadjusted	1 (ref)	−0.022 ± 0.008	−0.017 ± 0.008	0.009	0.029	0.049
Model 1	1 (ref)	−0.021 ± 0.009	−0.020 ± 0.008	0.021	0.017	0.027
Model 2	1 (ref)	−0.021 ± 0.009	−0.019 ± 0.008	0.020	0.020	0.031
Model 3	1 (ref)	−0.021 ± 0.009	−0.019 ± 0.008	0.019	0.021	0.033
Model 4	1 (ref)	−0.021 ± 0.009	−0.019 ± 0.008	0.019	0.021	0.033
Model 5	1 (ref)	−0.021 ± 0.009	−0.018 ± 0.008	0.020	0.026	0.042
	Occupational standing					
	Never or seldom	Sometimes	Often or always			
	*(n = 537)*	*(n = 1153)*	*(n = 1720)*	P1	P2	P-Trend
CIMT (mm)	0.716 ± 0.188	0.716 ± 0.182	0.712 ± 0.180			
Unadjusted	1 (ref)	0.000 ± 0.009	−0.004 ± 0.009	0.995	0.617	0.520
Model 1	1 (ref)	0.006 ± 0.010	0.005 ± 0.010	0.543	0.598	0.699
Model 2	1 (ref)	0.006 ± 0.010	0.005 ± 0.010	0.563	0.637	0.741
Model 3	1 (ref)	0.006 ± 0.010	0.005 ± 0.010	0.593	0.631	0.717
Model 4	1 (ref)	0.005 ± 0.010	0.005 ± 0.010	0.601	0.643	0.730
Model 5	1 (ref)	0.006 ± 0.010	0.004 ± 0.010	0.584	0.658	0.758

Data presented as mean ± standard deviation or unadjusted/adjusted mean difference compared to referent group (<2 h/day) ± standard error

P1 = Sometimes vs. Never of Seldom; P2 = Often or Always vs. Never or Seldom

*CIMT* carotid intima-media thickness

Model 1: Adjusted for age and sex

Model 2: Adjusted for covariates in model 1 plus education < high school, income < $50,000, fulltime employment, heavy alcohol drinking, current smoking, energy intake, history of myocardial infarction, and statin use

Model 3: Adjusted for covariates in model 2 plus diabetes, hypertension, BMI, total cholesterol, HDL cholesterol, LDL cholesterol, and estimated glomerular filtration rate <60 ml/min/1.73 m^2^

Model 4: Adjusted for covariates in model 3 plus levels of moderate to vigorous physical activity (poor, intermediate, or ideal)

Model 5: Adjusted for covariates in model 4 plus television viewing category (<2, 2–4, or >4 h/day)

### Occupational standing and CIMT

In unadjusted and adjusted models, occupational standing was not associated with CIMT (Table 3, Lower Panel).

## Discussion

In this community-based sample of African Americans, longer daily TV viewing, a leisure-time sedentary behavior, was associated with greater CIMT in adjusted models that included leisure-time MVPA level and CVD risk factors. In contrast, more frequent occupational sitting, a non-leisure time sedentary behavior, was associated with lower CIMT. These results suggest the association between sedentary behaviors and subclinical atherosclerosis in African Americans varies by leisure- and non-leisure types of sedentary behavior.

A meta-analysis of observational prospective studies has shown that TV viewing is associated with an increased risk of type 2 diabetes, fatal and non-fatal cardiovascular events, and all-cause mortality [28]. Our findings confirm the association of longer TV viewing with CVD risk and provide some of the first available data linking TV viewing to CIMT, a phenotype of early atherosclerosis. This finding may provide insight into one of the biologic pathways (i.e., early vascular changes) through which sedentary behavior may lead to CVD outcomes. Previous findings from a population-based study of 1778 white adults free of CVD risk factors who were enrolled in the National Heart, Lung, and Blood Institute Family Heart Study showed no significant association between daily TV viewing time and CIMT [29]. The null findings observed in the Family Heart Study could, in part, be attributed to the lower daily TV viewing time in the study sample, as the majority of participants (two-thirds) reported watching TV ≤2 h/day, a level that meta-analysis data suggest does not increase risk for some health outcomes [28]. In contrast, the level of TV viewing in the JHS sample was markedly higher as 71 % of participants watched TV ≥2 h/day. Racial differences in the study samples (white vs. African American) and the exclusion of participants with established CVD risk factors (diabetes, hypertension, and hypercholesterolemia) in the Family Heart Study may also be contributing factors to the divergent findings between the two studies.

The association between daily TV viewing and CIMT in the JHS sample also provides evidence to implicate TV viewing as a CVD risk factor among African Americans. These findings are consistent with a recent study by Matthews et al. which demonstrated an association between longer TV viewing and higher risk for all-cause mortality among 63,308 African Americans in the Southern Community Cohort Study [11]. In contrast, sedentary screen time (combined TV viewing and computer use) was not associated with left ventricular structure and function among 1327 young African Americans in the CARDIA study [8]. Similarly, a lack of association between accelerometer-measured sedentary time and CVD risk factors was reported among 835 African Americans in the NHANES survey [9]. Reasons for the discrepant findings could, in part, be attributed to differences in sample characteristics, CVD-related outcomes, and the type of sedentary behavior measured. Notably, it has been demonstrated that self-reported sedentary behavior, in particular self-reported TV viewing, is more consistently associated with CVD risk than objective measurements of sedentary behavior [30]. Between-study differences, however, should be interpreted cautiously when comparing TV viewing time to objectively-measured sedentary time as TV viewing represents one type of sedentary behavior in a single domain (leisure time) while objectively-measured sedentary time comprises behavior across all domains.

In the current study, the finding that frequent occupational sitting was associated with lower CIMT adds to a growing body of literature which has previously reported either a lack of or inverse association between occupational sitting and CVD risk. A systematic review showed that of 43 identified studies, 20 reported a null finding between occupational sitting and health outcomes/conditions and five reported a decreased risk with greater occupational sitting [31]. Similar to our discrepant findings for TV viewing and occupational sitting, data from the 1958 British Birth Cohort (~97 % whites and 3 % non-whites) showed differential associations of TV viewing and occupational sitting with 5 year gain in BMI. In that landmark study, higher levels of TV viewing were associated with greater positive gains in BMI, whereas more frequent occupational sitting was associated with a negative trend in BMI change [13]. Inconsistent associations for leisure-time sedentary behaviors and occupational sitting have also been reported in several other studies. Among 7660 middle-aged adults in the 1958 British Birth Cohort, higher levels of TV viewing, but not occupational sitting, had adverse associations with CVD biomarkers including C-reactive protein and fibrinogen [12]. In a Danish population-based study of 2544 adults, leisure-time sitting was adversely associated with cardio-metabolic risk factors including LDL cholesterol, cardiorespiratory fitness, and adiposity measures, while no significant associations were observed among these measures for occupational sitting [14, 32].

There are a number of possible explanations for the discrepant findings between TV viewing and occupational sitting. First, TV viewing may displace leisure-time MVPA [33, 34]. Second, the TV viewing-CVD risk association may be reflective of the lower energy expenditure of TV viewing in comparison to occupational sitting [25]. Third, the pattern of sedentary behavior may be different (e.g., prolonged, uninterrupted sedentary behavior [e.g., sitting for hours at a time] when watching TV versus more frequent and/or longer breaks from sedentary behavior at work). Finally, TV viewing is associated with increased energy-dense food and sugar-sweetened beverage consumption [35]. As laboratory-based studies have shown deleterious postprandial glucose responses during prolonged sedentary behavior, the timing of sedentary behaviors around energy-dense meals (such as during TV viewing) may be a contributing factor to the sedentary behavior-CVD risk association.

Reasons for our finding that more frequent occupational sitting is associated with lower CIMT are unclear but could, in part, be attributed to differences in white-collar vs. blue-collar work. Blue-collar workers, whom engage in less frequent occupational sitting than white-collar workers [36], consume a less healthy diet, have poorer sleep quality, and have a complex mix of work-related psychosocial factors (low job status, effort-reward imbalance, minimal health benefits, high job strain, hazardous work environment) that may contribute to the development of subclinical atherosclerosis [37, 38].

As the majority of daily sedentary time is accumulated in the workplace [39], there has been an emergence of consumer devices (sit-to-stand desks, treadmill workstations) to promote either standing or walking in the workplace as an alternative to sitting. Although the health benefits of physical activity are well established, limited empirical evidence supports standing as a means for interrupting periods of sedentary behavior. In the present study, more frequent occupational standing was not associated with lower CIMT. Occupational standing was also not associated with a reduced risk for obesity or type two diabetes in the Nurses’ Health Study [40]. In contrast, greater daily time spent standing (pooling leisure- and non-leisure domains) has been reported to be associated with a lower risk for CVD and all-cause mortality in a national cohort of Canadian adults [41]. Future studies are needed to confirm if standing renders cardiovascular benefits and whether any potential health benefits from standing vary by leisure and non-leisure domains.

There are several strengths to our study. First, the JHS is one of the largest community-based studies ever conducted among African Americans. This landmark study provided a unique opportunity to characterize a modifiable risk factor (sedentary behavior) in African Americans that may be amenable to behavioral intervention. Second, sedentary behaviors that occur in two different domains (leisure and non-leisure) were assessed, whereas many previous studies only focused on types of sedentary behavior in a single or combined domain. Finally, CIMT, an indicator of subclinical atherosclerosis, was measured by trained technicians using a standardized protocol with strict quality control procedures.

Several limitations must also be noted when interpreting our findings. First, sedentary behavior was measured by self-report. However, self-report questionnaires can provide information about sedentary behavior in specific domains (e.g., leisure- and non-leisure) which are not available from objectively measured data. Second, questions on TV viewing and occupational sitting had different response formats: for TV viewing, participants responded using pre-defined duration categories of hours/day or week (e.g., 1–2 h/day); for occupational sitting, participants responded using pre-defined frequency categories (e.g., ‘never’, ‘seldom’). Thus, the difference in measurement precision for assessing TV viewing and occupational sitting may have affected our study findings. Third, the JHS was conducted in a single metropolitan area in the Southeastern US, possibly limiting its generalizability to other African American populations. Fourth, although we controlled for many potentially confounding variables that could account for the discrepant findings between TV viewing and occupational sitting, there may be residual confounding from unmeasured factors. Finally, because of the cross-sectional nature of our analyses, we cannot infer causality.

## Conclusions

In this community-based sample of African Americans the association between sedentary behavior and subclinical atherosclerosis varied by TV viewing and occupational sitting. These data provide evidence that leisure-based sedentary behavior, in particular TV viewing, is associated with higher CIMT in African Americans. In contrast, more frequent occupational sitting was associated with lower CIMT in our study sample. Future research may be warranted to determine whether TV viewing is a potential target for behavioral intervention to mitigate CVD risk among African Americans. Elucidating factors that contribute to the differential associations of leisure and non-leisure types of sedentary behavior with CVD risk (e.g., Does the way sedentary behavior is patterned [sitting for hours at a time] carry any clinical significance beyond the total volume of sedentary time? Is the timing of sedentary behavior around energy-dense meals more harmful?) may also be warranted to inform future physical activity guidelines.

## Additional files

Additional file 1:
**Supplemental Methods.** Supplemental References. **Table S1.** Characteristics of JHS participants included and not included from analyses. **Table S2.** Characteristics of Jackson Heart Study participants (*n* = 3410) by category of occupational sitting. **Table S3.** Characteristics of Jackson Heart Study participants (*n* = 3410) by category of occupational standing. (DOCX 45 kb)
