# Supplementary material for: Sedentary behavior and subclinical atherosclerosis in African Americans: cross-sectional analysis of the Jackson heart study
Source: Int J Behav Nutr Phys Act. 2016 Mar 1;13:31. doi: 10.1186/s12966-016-0349-y (PMC4772318; doi:10.1186/s12966-016-0349-y)
Supplement: Additional file 1: — Supplemental Methods. Supplemental References. Table S1. Characteristics of JHS participants included and not included from analyses. Table S2. Characteristics of Jackson Heart Study participants (n = 3410) by category of occupational sitting. Table S3. Characteristics of Jackson Heart Study participants (n = 3410) by category of occupational standing. (DOCX 45 kb) [file 12966_2016_349_MOESM1_ESM.docx]

**ADDITIONAL FILE 1**

**Sedentary Behavior and Subclinical Atherosclerosis in African Americans: Cross-sectional Analysis of the Jackson Heart Study**

Keith M. Diaz^1^, John N. Booth III^2^, Samantha R. Seals^3^, Steven P. Hooker^4^, Mario Sims^5^, Patricia M. Dubbert^6^, Paul Muntner^2^, Daichi Shimbo^1^

^1^Center for Behavioral Cardiovascular Health, Department of Medicine, Columbia University Medical Center, New York, NY; ^2^Department of Epidemiology, School of Public Health, University of Alabama Birmingham, Birmingham, AL; ^3^Center of Biostatistics and Bioinformatics, University of Mississippi Medical Center, Jackson, MS; ^4^School of Nutrition and Health Promotion, Arizona State University, Phoenix, AZ; ^5^Department of Medicine, University of Mississippi Medical Center, Jackson, MS; ^6^South Central Mental Illness, Research, and Clinical Center and Little Rock Geriatric Research, Education, and Clinical Center, Little Rock , AR

**Author Emails:** KMD: kd2442@columbia.edu; JNB III: jnbooth@uab.edu; SRS: sseals@umc.edu; SPH: Steven.Hooker@asu.edu; MS: msims2@umc.edu; PMD: Patricia.Dubbert@va.gov; PM: pmunter@uab.edu; DS:ds2231@cumc.columbia.edu

**Author Correspondence:**

Keith M. Diaz

Columbia University Medical Center

622 West 168^th^ Street, PH9-319

New York, NY 10032

Tel #: 212-304-5231

Fax #: 212-305-3172

Email: [kd2442@columbia.edu](mailto:kd2442@columbia.edu)

**SUPPLEMENTAL METHODS**

**Covariates:** Sociodemographic characteristics (age, sex, education, income, employment status) selected CVD risk factors (BMI, diabetes, hypertension, cholesterol), lifestyle behaviors (cigarette smoking, alcohol drinking, energy intake, physical activity), estimated glomerular filtration rate (eGFR), history of myocardial infarction, and statin use were included as covariates.

Trained African American interviewers administered standard questionnaires to ascertain socioeconomic status information during the in-home visit. Education was measured as the highest level of schooling completed and classified into six categories: less than high school; high school diploma or graduate equivalency degree; vocational training or some college; associate’s degree; bachelor’s degree; and postgraduate degree. Annual family income was self-reported into 11 categories ranging from under $5,000 to $100,000 and above. Employment status was self-reported into the following categories: working full-time, working part-time, employed but temporarily laid off, sick or on leave, unemployed but looking for work, unemployed and not looking for work, homemaker, retired from usual job and not working, and retired from my usual job but working for pay.

BMI was calculated as the ratio of in-clinic measurements of weight (in kilograms) to height (in meters squared). Alcohol consumption was determined by a 5-item, interview-administered questionnaire that queried whether the participant had ever consumed alcohol and assessed quantity-frequency information about alcohol intake in the past 12 months. Heavy alcohol drinking was defined as drinking more than 14 drinks per week for men or more than 7 drinks per week for women. Current smoking status was defined as any participant who had smoked at least 400 cigarettes in their lifetime and was currently smoking at the time of their in-home visit. Energy intake was assessed using a 158-item food frequency questionnaire (FFQ) adapted from the Lower Mississippi Delta Nutrition Intervention Research Initiative FFQ that was designed for assessing diet of the US population living in the south [1]. Total energy intake was calculated from contributions of each of the 158 food items in the FFQ.

Medications taken within 2 weeks prior to the clinical examination were brought to the examination and transcribed verbatim with subsequent medication coding by a pharmacist using the Medispan dictionary and classified into categories according to the Therapeutic Classification System. Fasting blood samples were collected at the clinical examination according to standardized procedures, and the assessments of plasma glucose, lipids (total cholesterol, low density lipoprotein [LDL] cholesterol, and high density lipoprotein [HDL] cholesterol), and serum creatinine were processed at the Central Laboratory (University of Minnesota) as previously described [2]. The presence of diabetes mellitus was defined as one or more of the following: a measured fasting glucose of ≥126 mg/dL, a hemoglobin A1c ≥6.5%, or the use of hypoglycemic medications within 2 weeks of the clinic visit. eGFR was calculated using the Chronic Kidney Disease Epidemiology Collaboration (CKD-EPI) equation [3]. Seated blood pressure measurements were obtained at the clinical examination by trained staff with a Hawksley random-zero sphygmomanometer (Hawksley and Sons Ltd.) in the right arm after a 5-minute rest. Hypertension was defined as systolic blood pressure ≥140 mmHg, diastolic blood pressure ≥ 90 mmHg, or being on antihypertensive medication. History of myocardial infarction was defined as a self-reported history of health care provider-diagnosed myocardial infarction.

**SUPPLEMENTAL REFERENCES**

1. Carithers TC, Talegawkar SA, Rowser ML, Henry OR, Dubbert PM, Bogle ML, et al. Validity and Calibration of Food Frequency Questionnaires Used with African-American Adults in the Jackson Heart Study. *J Am Diet Assoc*. 2009;109(7):1184-93.
2. Carpenter MA, Crow R, Steffes M, Rock W, Heilbraun J, Evans G, et al. Laboratory, reading center, and coordinating center data management methods in the jackson heart study. *Am J Med Sci.* 2004;(3)328:131-44.
3. Levey AS, Stevens LA, Schmid CH, Zhang YL, Castro AF 3rd, Feldman HI, et al. A new equation to estimate glomerular filtration rate. *Ann Intern Med*. 2009;150(9):604–12.

| **Table S1.** Characteristics of JHS participants included and not included from analyses. | | | |
| --- | --- | --- | --- |
|  |  |  |  |
| **Variable** | **Included**  *(n=3410)* | **Not Included**  *(n=1591)* | **P-Value** |
| Age (years) | 53.5 ± 12.7 | 58.7 ± 12.4 | <0.001 |
| Male sex (%) | 37.3 | 35.1 | 0.114 |
| Education < HS (%) | 17.3 | 25.8 | <0.001 |
| Income < $50,000 (%) | 69.1 | 74.8 | <0.001 |
| Fulltime Employment (%) | 55.2 | 39.0 | <0.001 |
| Heavy alcohol drinking (%)^a^ | 3.8 | 3.0 | 0.155 |
| Current Smoking (%) | 12.9 | 13.8 | 0.371 |
| Energy Intake (kcal/d) | 2224.8 ± 1273.2 | 2077.5 ± 1185.1 | <0.001 |
| History of MI (%) | 4.8 | 6.7 | 0.004 |
| Statin Use (%) | 11.0 | 14.5 | <0.001 |
| Diabetes (%)^b^ | 19.6 | 26.3 | <0.001 |
| Hypertension (%)^c^ | 58.6 | 66.5 | <0.001 |
| BMI (kg/m^2^) | 31.8 ± 7.3 | 31.6 ± 7.1 | 0.308 |
| Obese (%) | 53.7 | 52.7 | 0.751 |
| Total Cholesterol (mg/dL) | 199.1 ± 40.5 | 199.6 ± 39.4 | 0.677 |
| HDL Cholesterol (mg/dL) | 51.6 ± 14.5 | 52.1 ± 14.9 | 0.252 |
| LDL Cholesterol (mg/dL) | 127.0 ± 37.0 | 126.0 ± 35.9 | 0.344 |
| eGFR <60 ml/min/1.73 m^2^ (%) | 5.6 | 8.1 | 0.001 |
| Leisure-time MVPA (min/week) | 70.1 ± 113.6 | 58.4 ± 104.0 | <0.001 |
| Level of MVPA (%)^d^ |  |  | <0.001 |
| Poor | 46.9 | 53.7 |  |
| Intermediate | 32.4 | 30.2 |  |
| Ideal | 20.7 | 16.1 |  |
| Data presented as mean ± standard deviation or percentage. | | | |
| HDL, high density lipoprotein; HS, high school; MI, myocardial infarction; MVPA, moderate to vigorous physical activity. | | | |
| ^a^Defined as >14 drinks/week for men; >7 drinks/week for women. | | | |
| ^b^Defined as fasting glucose ≥126 mg/dL, HbA1c ≥6.5%, or use of diabetic medication. | | | |
| ^c^Defined as blood pressure ≥140/90 mmHg and/or use of antihypertensive medication. | | | |
| ^d^Defined according to American Heart Association Life’s Simple 7 criteria for minutes/week of moderate or vigorous physical activity. Poor physical activity: 0 minutes/week of leisure-time moderate or vigorous physical activity. Intermediate physical activity: >0 and <150 minutes/week of leisure-time moderate physical activity; and >0 and <75 minutes/week of leisure-time vigorous physical activity. Ideal physical activity: ≥150 minutes/week of leisure-time moderate physical activity; or ≥75 minutes/week of leisure-time vigorous physical activity. | | | |

| **Table S2.** Characteristics of Jackson Heart Study participants (n=3,410) by category of occupational sitting. | | | | |
| --- | --- | --- | --- | --- |
|  | **Occupational Sitting** | | |  |
| **Variable** | **Never or Seldom**  *(n=945)* | **Sometimes**  *(n=988)* | **Often or Always**  *(n=1477)* | **P-Trend** |
| Age (years) | 54.4 ± 12.5 | 53.6 ± 12.4 | 52.9 ± 13.1 | 0.018 |
| Male sex (%) | 38.5 | 36.8 | 36.8 | 0.409 |
| Education < HS (%) | 19.6 | 16.5 | 16.4 | 0.054 |
| Income <$50,000 (%) | 69.7 | 69.2 | 68.5 | 0.519 |
| Fulltime Employment (%) | 52.1 | 54.7 | 57.7 | 0.006 |
| Heavy alcohol drinking (%)^a^ | 3.7 | 3.7 | 3.9 | 0.843 |
| Current Smoking (%) | 14.8 | 11 | 12.9 | 0.272 |
| Energy Intake (kcal/d) | 2251.2 ± 1321.9 | 2244.3 ± 1292.2 | 2195.2 ± 1228.3 | 0.497 |
| History of MI (%) | 5.5 | 4.9 | 4.3 | 0.162 |
| Statin Use (%) | 13.4 | 10.8 | 9.7 | 0.006 |
| Diabetes (%)^b^ | 20.3 | 19.8 | 19 | 0.402 |
| Hypertension (%)^c^ | 58.6 | 58.9 | 58.4 | 0.893 |
| BMI (kg/m^2^) | 31.8 ± 7.5 | 31.7 ± 7.1 | 31.9 ± 7.4 | 0.731 |
| Obese (%) | 52.0 | 54.5 | 54.2 | 0.289 |
| Total Cholesterol (mg/dL) | 200.9 ± 39.6 | 199 ± 40.9 | 198.1 ± 40.8 | 0.291 |
| HDL Cholesterol (mg/dL) | 51.9 ± 14.7 | 51.5 ± 14.4 | 51.5 ± 14.4 | 0.771 |
| LDL Cholesterol (mg/dL) | 128.1 ± 36.8 | 127.5 ± 38.2 | 126 ± 36.3 | 0.402 |
| eGFR <60 ml/min/1.73 m^2^ (%) | 4.3 | 7.2 | 5.3 | 0.477 |
| Leisure-time MVPA (min/week) | 68.2 ± 110.4 | 68.9 ± 113.9 | 72.2 ± 115.5 | 0.643 |
| Level of MVPA (%)^d^ |  |  |  | 0.261 |
| Poor | 47.1 | 48.1 | 45.9 |  |
| Intermediate | 32.9 | 32.1 | 32.2 |  |
| Ideal | 19.9 | 19.8 | 21.9 |  |
| Data presented as mean ± standard deviation or percentage. | | | | |
| HDL, high density lipoprotein; HS, high school; MI, myocardial infarction; MVPA, moderate to vigorous physical activity. | | | | |
| ^a^Defined as >14 drinks/week for men; >7 drinks/week for women. | | | | |
| ^b^Defined as fasting glucose ≥126 mg/dL, HbA1c ≥6.5%, or use of diabetic medication. | | | | |
| ^c^Defined as blood pressure ≥140/90 mmHg and/or use of antihypertensive medication. | | | | |
| ^d^Defined according to American Heart Association Life’s Simple 7 criteria for minutes/week of moderate or vigorous physical activity. Poor physical activity: 0 minutes/week of leisure-time moderate or vigorous physical activity. Intermediate physical activity: >0 and <150 minutes/week of leisure-time moderate physical activity; and >0 and <75 minutes/week of leisure-time vigorous physical activity. Ideal physical activity: ≥150 minutes/week of leisure-time moderate physical activity; or ≥75 minutes/week of leisure-time vigorous physical activity. | | | | |

| **Table S3.** Characteristics of Jackson Heart Study participants (n=3,410) by category of occupational standing. | | | | |
| --- | --- | --- | --- | --- |
|  | **Occupational Standing** | | |  |
| **Variable** | **Never or Seldom**  *(n=537)* | **Sometimes**  *(n=1153)* | **Often or Always**  *(n=1720)* | **P-Trend** |
| Age (years) | 53.4 ± 13.3 | 53.4 ± 12.6 | 53.7 ± 12.7 | 0.770 |
| Male sex (%) | 41.2 | 37.2 | 36.1 | 0.049 |
| Education < HS (%) | 18.7 | 16.5 | 17.5 | 0.775 |
| Income <$50,000 (%) | 69.6 | 68.6 | 69.2 | 0.960 |
| Fulltime Employment (%) | 54.0 | 55.8 | 55.3 | 0.729 |
| Heavy alcohol drinking (%)^a^ | 4.4 | 3.3 | 4.0 | 0.993 |
| Current Smoking (%) | 14.6 | 11.9 | 13.0 | 0.616 |
| Energy Intake (kcal/d) | 2224.4 ± 1183.0 | 2158.4 ± 1191.6 | 2269.3 ± 1349.8 | 0.079 |
| History of MI (%) | 5.8 | 4.8 | 4.5 | 0.250 |
| Statin Use (%) | 10.8 | 8.9 | 12.5 | 0.060 |
| Diabetes (%)^b^ | 20.3 | 19.3 | 19.5 | 0.774 |
| Hypertension (%)^c^ | 59.8 | 58.1 | 58.5 | 0.735 |
| BMI (kg/m^2^) | 32.3 ± 7.4 | 31.6 ± 6.7 | 31.8 ± 7.7 | 0.149 |
| Obese (%) | 54.9 | 53.6 | 53.3 | 0.178 |
| Total Cholesterol (mg/dL) | 197.8 ± 42.7 | 199.8 ± 41 | 199.1 ± 39.4 | 0.647 |
| HDL Cholesterol (mg/dL) | 50.6 ± 14.7 | 51.4 ± 13.6 | 52.1 ± 15 | 0.124 |
| LDL Cholesterol (mg/dL) | 125.1 ± 36.1 | 128.3 ± 38.1 | 126.8 ± 36.5 | 0.274 |
| eGFR <60 ml/min/1.73 m^2^ (%) | 5.7 | 6.0 | 5.3 | 0.559 |
| Leisure-time MVPA (min/week) | 75.3 ± 119.4 | 67.6 ± 111.7 | 70.2 ± 113.1 | 0.435 |
| Level of MVPA (%)^d^ |  |  |  | 0.911 |
| Poor | 47.3 | 48.6 | 45.6 |  |
| Intermediate | 29.1 | 31.7 | 33.9 |  |
| Ideal | 23.6 | 19.8 | 20.5 |  |
| Data presented as mean ± standard deviation or percentage. | | | | |
| HDL, high density lipoprotein; HS, high school; MI, myocardial infarction; MVPA, moderate or vigorous physical activity. | | | | |
| ^a^Defined as >14 drinks/week for men; >7 drinks/week for women. | | | | |
| ^b^Defined as fasting glucose ≥126 mg/dL, HbA1c ≥6.5%, or use of diabetic medication. | | | | |
| ^c^Defined as blood pressure ≥140/90 mmHg and/or use of antihypertensive medication. | | | | |
| ^d^Defined according to American Heart Association Life’s Simple 7 criteria for minutes/week of moderate or vigorous physical activity. Poor physical activity: 0 minutes/week of moderate or vigorous physical activity. Intermediate physical activity: >0 and <150 minutes/week of moderate physical activity; and >0 and <75 minutes/week of vigorous physical activity. Ideal physical activity: ≥150 minutes/week of moderate physical activity; or ≥75 minutes/week of vigorous physical activity. | | | | |
